# Metastasis Model of Cancer Stem Cell-Derived Tumors

**DOI:** 10.3390/mps3030060

**Published:** 2020-08-21

**Authors:** Hager Mansour, Ghmkin Hassan, Said M. Afify, Ting Yan, Akimasa Seno, Masaharu Seno

**Affiliations:** 1 Department of Medical Bioengineering, Graduate School of Natural Science and Technology, Okayama University, Okayama 700-8530, Japan; pcnp49mj@s.okayama-u.ac.jp (H.M.); aseno@okayama-u.ac.jp (A.S.); 2 Laboratory of Nano-Biotechnology, Graduate School of Interdisciplinary Science and Engineering in Health Systems, Okayama University, Okayama 700-8530, Japan; pthz2c4o@s.okayama-u.ac.jp (G.H.); saidafify@s.okayama-u.ac.jp (S.M.A.); 3 Department of Microbiology and Biochemistry, Faculty of Pharmacy, Damascus University, Damascus 10769, Syria; 4 Division of Biochemistry, Chemistry Department, Faculty of Science, Menoufia University, Shebin El Koum-Menoufia 32511, Egypt; 5 Department of Pathology, Shanxi Key Laboratory of Carcinogenesis and Translational Research on Esophageal Cancer, Shanxi Medical University, Taiyuan 030001, China; enntei@hotmail.com

**Keywords:** metastasis, cancer stem cells, transplantation, surgery

## Abstract

Metastasis includes the dissemination of cancer cells from a malignant tumor and seed in distant sites inside the body forming secondary tumors. Metastatic cells from the primary tumor can move even before the cancer is detected. Therefore, metastases are responsible for more than 90% of cancer-related deaths. Over recent decades there has been adequate evidence suggesting the existence of CSCs with self-renewing and drug-resistant potency within heterogeneous tumors. Cancer stem cells (CSCs) act as a tumor initiating cells and have roles in tumor retrieve and metastasis. Our group recently developed a unique CSC model from mouse induced pluripotent stem cells cultured in the presence of cancer cell-conditioned medium that mimics tumors microenvironment. Using this model, we demonstrated a new method for studying metastasis by intraperitoneal transplantation of tumors and investigate the metastasis ability of cells from these segments. First of all, CSCs were injected subcutaneously in nude mice. The developed malignant tumors were minimized then transplanted into the peritoneal cavity. Following this, the developed tumor in addition to lung, pancreas and liver were then excised and analyzed. Our method showed the metastatic potential of CSCs with the ability of disseminated and moving to blood circulation and seeding in distant organs such as lung and pancreas. This method could provide a good model to study the mechanisms of metastasis according to CSC theory.

## 1. Introduction

Metastasis is spreading of cancer to tissues or organs far from their original sites where they originated. Cancer metastasis enables forming secondary tumors in distant organs and is major responsible for the mortality and morbidity of cancer [1].

The metastasis includes several events beginning with dissemination of cancer cells from tumors, invading stroma, intravasation and seeding in secondary sites where they form metastasis [2]. Many genes are changing during these stages changing cell phenotypes. Genes, such as E-cadherin, slug and twist, contribute to give cancer cells the dissemination and movement ability driving metastasis events [3].

On the other hand, cancer stem cells (CSCs) represent the subpopulation of cancer cells with the ability to differentiate into other cell phenotypes and initiated tumorigenesis. CSCs were proved to have essential roles in metastasis and drug resistance characters of cancer cells. When a small number of CSCs are injected into immunocompromised animal model, they can form new tumors [4]. CSCs also express stemness markers, Nanog, Sox2 and Oct3/4, and CSC markers such as EpCAM, CD133, CD44, and CD24. CSCs usually enrichment from either from cancer cell lines or from patient derived samples. Isolation of CSC is still considered a challenging and demanding procedure [5,6]. Induced pluripotent stem cells (iPSCs) have opened the door for personalized medicine and facilitated modeling a wide range of diseases. Our lab has established novel CSC models by converting iPSCs into CSCs. Conversion of iPSCs into CSCs has been demonstrated by culturing iPSCs in the presence of conditioned media (CM) from different cancer cell lines secreting cytokines, chemokines and growth factors that direct the conversion without genetic manipulation of iPSCs. Accordingly, we successfully established different mouse cell models using CM from lung, breast, pancreas and liver cancer cell lines [7,8,9,10,11].

The selection of the method to investigate the metastasis is critical for the identification and candidate genes and mechanisms that may regulate metastasis and for the evaluation of anti-metastatic drugs. Recent methods include detecting metastasis after transplantation of cancer cells or tissue either orthotopically or ectopically in addition to the injection of cancer cells in blood circulation or intraperitoneally [12,13]. However, these methods still do not accurately present the metastasis events especially regarding the disposition of cells from original tumors and injections cancer cells neglects dissemination step which is main the step in the metastasis events. Therefore, developing new methods is becoming important to investigate metastasis and screening new drugs. Our present unique models that enabling investigation of tumor progression events according to CSC theory. In this manner, we present here a new method for studying metastasis using CSC model developed from iPSCs. Our method investigates the ability of CSCs to disseminate from bulk intraperitoneally transplanted tumor and metastasis into secondary sites.

## 2. Experimental Design

**Figure 1:**
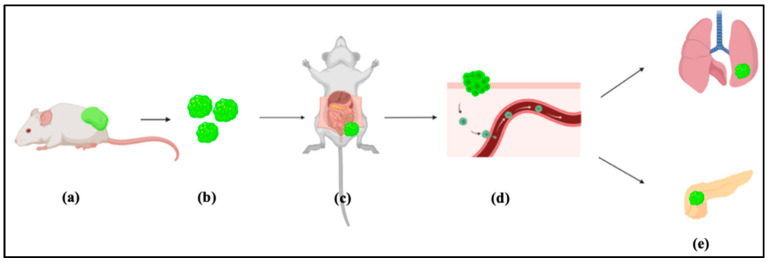
Representative scheme of the metastatic procedure using transplantation method. (**a**) Injected Cancer stem cells (CSCs) subcutaneously and leave it for 4 weeks to form malignant tumor. (**b**) Excised and minimized tumor. (**c**) Transplantation of the minimized malignant tumor in the in the peritoneal cavity. (**d**) CSCs disseminated through blood vessels. (**e**) Disseminated CSCs reached the organs and colonized.

We have developed a protocol for tumor tissue transplantation using cancer stem cell developed tumor. In our assay, we describe step by step evaluating the model of metastasis by tissue transplantation of CSC derived tumor as summarized in Figure 1. This protocol will be very important to guide researchers who will follow developing metastasis from CSCs to evaluate treatment strategies and molecular mechanisms of metastasis development from the sight of CSC theory. The surgical procedure is performed under sterile conditions. All instruments are sterilized by autoclaving before the procedure.

### 2.1. Materials

- Dulbecco’s Modified Eagle Medium (DMEM; Thermo Fisher Scientific, cat. no. 31966).
- Minimum Essential Media MEM non-essential amino acid solution (100×) (Wako, Osaka, Japan, catalog number: 139-15651).
- Fetal bovine serum (FBS, Gibco, Life Technologies, Massachusetts, MA, USA, Cat. No: 10437-028).
- Penicillin/streptomycin mixed solution (Nacalai Tesque, Kyoto, Japan, Cat. No-26253-84).
- L-glutamine (Nacalai Tesque, Kyoto, Japan, ((catalog number: 16948-04)).
- Trypsin, 0.25% Ethylenediamine tetraacetic acid (EDTA) (Atlanta Biologicals, Flowery Branch, GA, USA; Cat. no.: B81310).
- Phosphate buffered saline (PBS) (Genesee Scientific, El Cajon, CA, USA; Cat. no.: 25-508).
- Hank’s balanced salt solution (HBSS).
- Iodine solution (7.5% (wt/vol); Medline, cat. no. MDS093908).
- Isoflurane (100% (wt/wt); IsoFlo; Abbott, cat. no. B506).
- KnockOut^TM^ Serum Replacement (Gibco, NY, USA; Cat. No.: 10828028).
- Collagenase (Gibco, NY, USA; Cat. No.: 17018029/).
- Mouse induced pluripotent stem cells (miPSCs) (iPS-MEF-Ng-20D-17, Lot No. 012, Riken Cell Bank, Tokyo, Japan), in which puromycin (puro) resistant gene and green fluorescent protein (GFP) gene were cloned under the control of Nanog promoter.
- BALB/c-nu/nu immunodeficient mice, female, four weeks old (Charles River laboratories, kanagawa, Japan).
- 70% ethanol (Sigma-Aldrich; Cat. No.: 459836-2).
- CaCl_2_ (Sigma-Aldrich; Cat. No.: C5670).
- Leukemia inhibitory factor, 1000 U/mL (LIF, Merk Millipore).

### 2.2. Equipment

- Sanyo MCO-19AIC(UV) CO_2_ incubator (Marshall Scientific, Hampton, WV, USA).
- Labculture^®^ Class II, Type A2 Biological Safety Cabinets (E-Series).
- Olympus IX81 microscope (Olympus, Tokyo, Japan).
- Laser scanning confocal microscope, FV-1000, Olympus, Tokyo, Japan.
- Tissue culture-treated plate, 60 mm dish (TPP Techno Plastic Products AG Schaffhausen, Switzerland, Cat. No 93060).
- Tissue culture-treated plate, 100 mm dish (TPP Techno Plastic Products AG Schaffhausen, Switzerland, Cat. No 93100).
- Filter max 250 mL (TPP, Switzerland, Cat. No 99255).
- Falcon^®^ Conical Centrifuge Tubes (15 mL; BD Falcon, New York, NY, USA Cat. No 352095).
- Falcon^®^ Conical Centrifuge Tubes (50 mL; BD Falcon, New York, NY, USA Cat. No 352070).
- Liquid N2 storage tank.
- Inverted microscope with bright field (Nikon, model no. DIAPHOT 200).
- Automated cell counter (TC20; Bio-Rad Laboratories, cat. no. 1450102).
- 1.5-mL microcentrifuge tubes (Eppendorf, cat. no. 0030120086).
- Refrigerated centrifuge (Eppendorf, cat. no. 5810 R).
- 100-mm dishes (tissue culture treated; Corning, cat. no. 353003).
- Anesthesia machine (Vet Tech Solutions).
- Microcentrifuge 1.5-mL tubes (Eppendorf, cat. no. 0030120086).
- 5/10/25-mL plastic disposable pipette.

## 3. Procedure

- Cancer stem cell induction for in vivo injection.

Recently Yan et al., 2014, converted iPSCs into cancer stem cells in the presence of extracellular vesicles from Lewis lung carcinoma cell lines named miPS-LLCev cells. The converted cells showed self-renewal, differentiation and tumorigenic potential. Moreover, the primary culture cells sustain the expression of self-renewal and CSCs markers [14,15]. The detailed protocol of generating CSCs from iPSCs described by Afify et al. 2019 [16].

**Figure 2:**
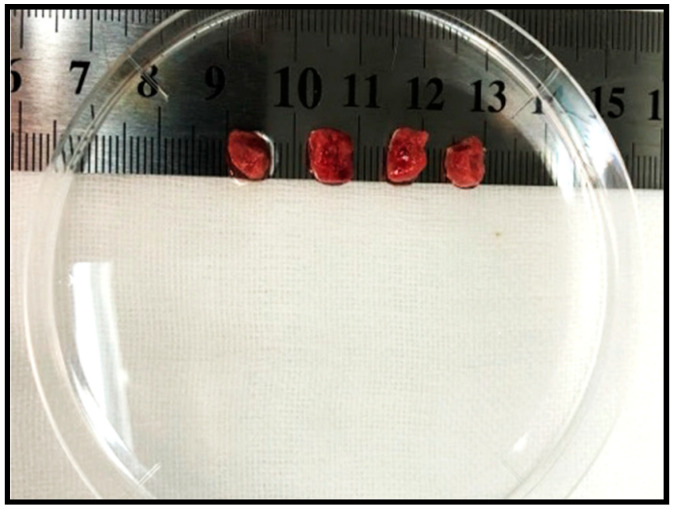
Fresh surgically resected mouse malignant tumor tissue ready to be transplanted.

II. CSCs preparation for injection
  - Revive miPS-LLCev cells on gelatin coated dish.
  - Change medium every 2 days.
  - When cells are 70% confluent, trypsinize the cells.
  - Collect cells from culturing flask and transfer it to 15 mL tube.
  - Spin down cells at 1000 rpm for 5 min.
  - Aspirate supernatant media.
  - Wash cells with sterile PBS.
  - Add PBS to tube, and again spin down cells at 1000 rpm for 5 min.
  - Aspirate supernatant PBS.
  - Resuspend cell pellet with 1 mL sterile BSS.
  - Count the cells.
  - Suspend 10^6^ cells in 100 μL sterile HBSS.
  - Keep the Eppendorf in ice.

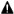
 **CRITICAL STEP:** draw cells into the syringe without a needle to prevent cell shearing.
  - Before injecting, flick or invert the syringe to ensure the cells are in suspension.
  - Inject cells slowly subcutaneously.
  - Four weeks later, malignant tumor should be observed
  - Excise and minimize the tumor tissue for intraperitoneal transplantation (Figure 2).

**Figure 3:**
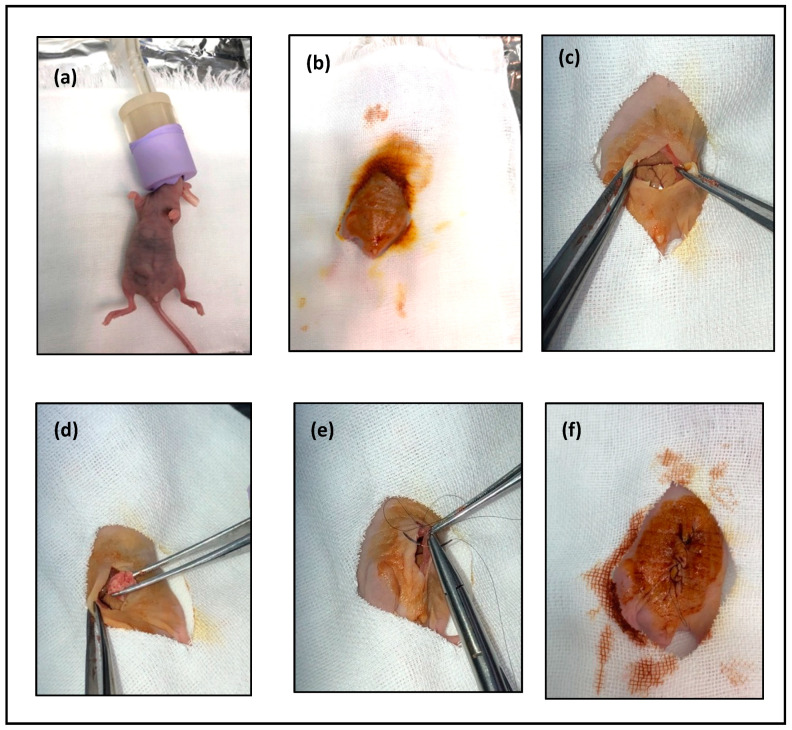
Transplantation method steps: (**a**) Anesthetize mouse. (**b**) Preparing the area for transplantation. (**c**) A 15-mm vertical midline incision through the skin. (**d**) Inserting the tumor tissue. (**e**), (**f**) Closing the abdominal wall and skin opening.

III. Intraperitoneal (IP) transplantation
  - Anesthetize mouse with 2% isoflurane (Figure 3a).
  - After the mouse is anesthetized, prepare the area for transplantation with 70% alcohol (Figure 3b).
  - Using the curved iris forceps, hold the skin and make a 15-mm vertical midline incision through the skin using the 24-mm iris scissors (Figure 3c).
  - Insert 1 mm of the tumor tissue intraperitoneal (Figure 3d).

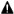
 **CRITICAL STEP:** try to avoid damaging any organ in the abdomen.
  - Close the abdominal wall and skin opening, performing continuous stitching (Figure 3e,f).
  - The day after surgery, check on the animal to make sure that the sutures are still correctly in place.
  - After four weeks, euthanatize mice with 5% of isoflurane through inhalation to ensure rapid loss of consciousness and respiratory and cardiac arrest followed by cervical dislocation to ensure the death of mice.

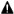
 **CRITICAL STEP: four weeks are mandatory so that the cells will have enough time to be disseminated from the original tumor to the other organs. Before four weeks, metastasis will not be visible enough.**
  - The mouse allografts were excised and cut into small pieces (approximately 1 mm^3^).
  - Wash in the PBS for three times.
  - Transfer the pieces into a 15-mL tube with 4 mL of dissociation buffer.
  - Incubate at 37 °C for 40 min.
  - Add 5 mL of DMEM containing 10% FBS to terminate the digestion.
  - Transfer suspended cells into new tubes.
  - Centrifuge at 1000 rpm for 5 min at 20 °C.
  - Resuspend the cell pellet in 5 mL PBS.
  - Centrifuge at 1000 rpm for 5 min 20 °C.
  - Aspirate PBS.
  - Resuspend the cell pellet in 5 mL 5 mL DMEM containing 10% FBS without LIF.
  - Seed 5 × 10^4^ cells into a 60 mm dish.
  - Treat the cells with puromycin for 24 h to remove the host cells.

## 4. Expected Results

**Figure 4:**
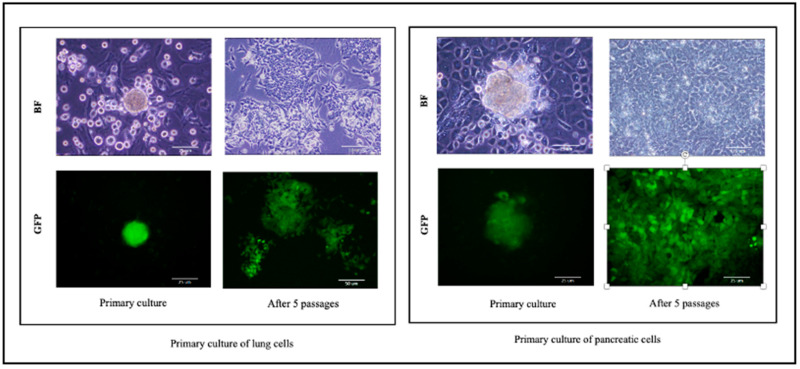
Primary cell cultures of metastatic tumors in pancreatic and lung. BF, bright field; GFP, detection of green fluorescence.

This protocol describes a technique of disposition of CSCs from primary tumor and dissemination to another organs which is main the step in the metastasis events. Our present unique models enable investigation of tumor progression events according to CSC theory. In this manner, we present here a new method for studying metastasis using CSC model developed from iPSCs. Our method investigates the ability of CSCs to disseminate from bulk of the intraperitoneally transplanted tumor through blood stream and metastasis into secondary sites. Therefore, developing new methods is becoming important to investigate metastasis steps clearly and screening of new drugs. In Figure 4, we showed the presence of CSCs in the primary cell culture by expressing GFP and cell morphology was observed and photographed using Olympus IX81 microscope equipped with a light fluorescence device.

**Figure 5:**
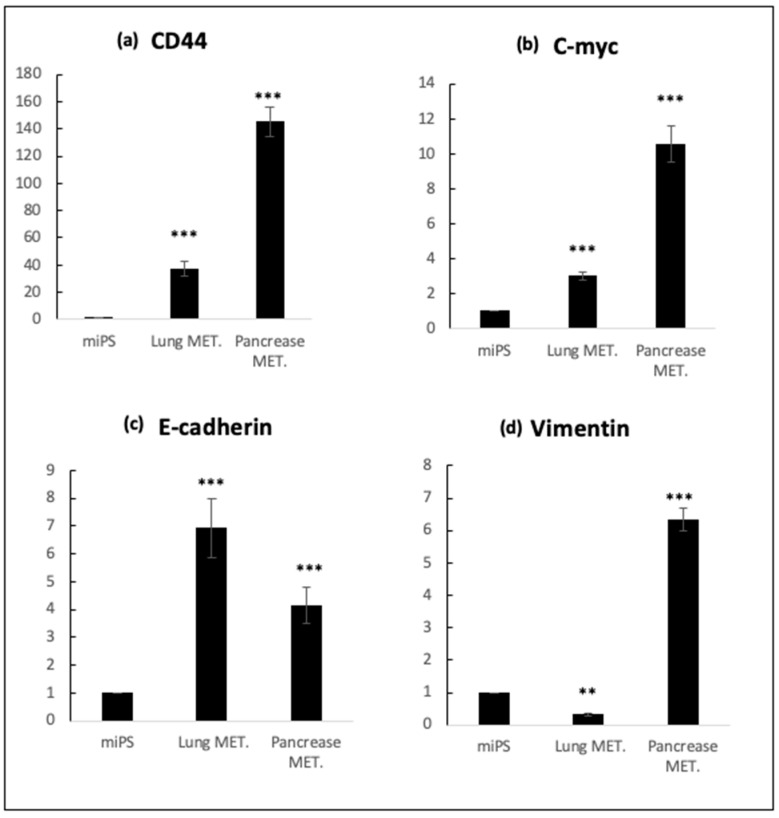
(**a**) RT-qPCR analysis of CSC marker CD44; (**b**) RT-qPCR analysis of stemness marker c-Myc (**c**), (**d**) RT-qPCR analysis of metastatic markers E-cadherin and vimentin in cells derived from intraperitoneal transplantation (lung and pancreatic metastatic cells) in comparison with miPS cells. MET.; Metastasis. (**, *p* ≤ 0.01; ***, *p* ≤ 0.001)

On the other hand, our result has been confirmed by RT-qPCR analysis, lung and pancreatic metastatic cells were confirmed to sustain the expression of endogenous stemness marker c-Myc, as much as miPSCs. On the other hand, the expression of CSC-marker CD44 was extremely elevated in both types of metastatic cells. Furthermore, the expression of metastatic markers vimentin and E-cadherin was significantly different between lung and pancreatic metastatic cells, whereas E-cadherin expression showed significantly higher expression of more than double when compared to miPSCs cells (*p* < 0.001). At the same time, vimentin showed relatively higher expression in pancreatic metastatic cells in comparison with miPSCs (Figure 5).

## 5. Reagents Setup

### Dissociation Buffer

Prepared in PBS containing:

- 0.25% trypsin
- 0.1% collagenase
- 20% KnockOut^TM^ Serum Replacement (Gibco, NY, USA).
- 1 mM of CaCl_2_.
